# Mutations in the notch signalling pathway are associated with enhanced anti‐tumour immunity in colorectal cancer

**DOI:** 10.1111/jcmm.15867

**Published:** 2020-09-14

**Authors:** Fei Wang, Jie Long, Liang Li, Zhi‐bin Zhao, Fang Wei, Yuan Yao, Wen‐Jing Qiu, Zi‐Xin Wu, Qing‐Qing Luo, Wei Liu, Yi‐Bo Quan, Zhe‐Xiong Lian, Jie Cao

**Affiliations:** ^1^ Department of General Surgery Guangzhou Digestive Disease Center Guangzhou First People's Hospital School of Medicine, South China University of Technology, Guangzhou Guangdong China; ^2^ Chronic Disease Laboratory Institutes for Life Sciences South China University of Technology Guangzhou China; ^3^ Institute of Immunology and School of Life Sciences University of Science and Technology of China Hefei China; ^4^ Department of General Surgery Guangzhou Digestive Disease Center Guangzhou First People's Hospital Guangzhou Medical University Guangzhou China

**Keywords:** anti‐tumour immunity, colorectal cancer, mutation, notch signalling pathway, tumour microenvironment

## Abstract

The Notch signalling pathway is involved in the development of several cancers, including colorectal cancer (CRC). However, whether mutations in this pathway could alter the CRC immunophenotype remains unknown. Here, we investigated the relationship between Notch signalling pathway mutations and the tumour immune microenvironment by analysing gene expression data from the GSE108989 single T cell RNA sequencing data set and The Cancer Genome Atlas (TCGA) data set. We found that Notch signalling pathway mutations were associated with an increased number of tumour‐specific CD8^+^ T cells and decreased number of inhibitory regulatory T (Treg) cells, representing an enhanced anti‐tumour response in the GSE108989 data set. In TCGA data set, we also found that Notch signalling pathway mutations were associated with enrichment of genes associated with immune activation pathways and higher expressions of *PDCD1*, *GZMB* and *PRF1*. Although Notch signalling pathway mutations did not affect the overall survival and disease‐free survival of CRC patients, they were associated with earlier disease stages and lower rates of metastasis. These results demonstrated that Notch signalling pathway mutations can enhance anti‐tumour immunity in CRC, as validated by the two data sets, suggesting that they may be promising biomarkers for immune checkpoint blockade therapies for CRC patients.

## 1 INTRODUCTION

Colorectal cancer (CRC) is one of the most common malignant tumours globally. It is estimated that there were more than 1.8 million new cases of CRC and 881 000 deaths worldwide in 2018, accounting for nearly 1 in 10 cancer cases and deaths.^1^

It has been reported that deficient mismatch repair (dMMR) and microsatellite instability (MSI) CRCs are associated with higher‐level immune infiltration^2^ and that they respond better to immune checkpoint blockades compared with those CRC patients without MSI.^3^ Some somatically mutated genes resulting in amino acid changes can generate immunogenic peptides (called neoantigens) that can participate in tumour‐specific immune responses in CRC.^4^ These results indicate that a biologically and therapeutically significant interplay exists between gene mutations and the immune microenvironment.

The highly conserved Notch signalling pathway modulates a series of fundamental cellular functions, including cell fate decision, maintenance of stemness, proliferation and apoptosis.^5^ Mammals have four NOTCH receptors (NOTCH 1‐4).^6^ The Notch signalling pathway is activated when the ligand expressed on the surface of one cell interacts with the receptor expressed on the plasma membrane of another cell.^6^ Following interaction with its ligand, the NOTCH receptor is cleaved by ADAM10/ADAM17 (S2 cleavage) and γ‐secretase complex (S3 cleavage). Following the S3 cleavage, the NOTCH intracellular domain (NICD) is released and translocates into the nucleus where it modulates the transcription of its target genes. Notch signalling is tightly regulated. For example, Fbxw7 (encoded by the *FBXW7* gene), which is the substrate‐recognition component of E3 ubiquitin ligase complex, can mediate NICD degradation via ubiquitination.^6^ NUMB is another well‐known inhibitor of the Notch signalling pathway, which can antagonize Notch signals through direct interaction with Notch ankyrin (ANK)‐repeats.^7^, ^8^, ^9^

In this study, we analysed the GSE108989 data set of T cells and found that *NOTCH1* and *FBXW7* mutations in CRC patients were associated with higher expression levels of *PDCD1*, *CTLA4* and *GZMB* of tumour‐infiltrating CD8^+^ T cells. These mutations could also decrease the infiltration of *EBI3* and *CD83* expressing Treg cells. Analysis of the TCGA CRC data set indicated that Notch signalling pathway mutations were associated with higher expression levels of *PDCD1*, *CTLA4* and *CD274* in CRC patients. These findings suggest that mutations in the Notch signalling pathway could modulate the immune microenvironment of CRC, which may indicate a better response to immune checkpoint blockades.

## 2 MATERIALS AND METHODS

### 2.1 GSE108989 CRC single T cell sequencing data set

GSE108989 data set was obtained from Gene Expression Omnibus (GEO) database. The data processing methods were described in the original article.^10^ Somatic mutation profile of these patients was obtained from supplementary materials of the original article.^10^

### 2.2 Analysis of GSE108989 data set

On acquiring the data set, we excluded the cells in which the number of unique genes was more than 6000 or less than 800. Only genes expressed in 10 or more cells were used for further analysis. We also disregarded the cells if their mitochondrial gene percentages were over 10%. Eventually, we obtained a data matrix of 23 370 genes and 11 138 cells. The matrix was normalized using the method LogNormalize in Seurat package, and the scale factor was set to the default value of 10 000. We performed dimensionality reduction analysis using the Seurat R package (version 3.1.1), which was also used in Zhang's original article.^10^ Firstly, we identified 2000 highly variable genes using the FindVariableFeatures function, and the selected method was ‘vst’. Then, we performed principal component analysis (PCA) using the variable genes and determined significant principal components (PCs) based on the ElbowPlot function from the Seurat package. The top 15 PCs were selected for t‐Distributed Stochastic Neighbor Embedding (t‐SNE) analysis. To cluster cells, we used the FindClusters function and set the resolution parameter at 0.1. Then, the CD4^+^ Foxp3^‐^ T cell, CD4^+^ Foxp3^+^ Treg and CD8^+^ T cell clusters were separated for further analysis.

### 2.3 CRC TCGA data set analysis

In order to validate the differences in T cell subtype composition between patients with Notch pathway mutation and non‐mutation derived from the CRC GSE108989 data set, we used the TCGA CRC data set to confirm the gene expression signatures between CRC patients with or without Notch pathway mutations. Gene expression (reads per kilobase per million, RPKM normalized), whole exome sequencing and clinicopathological data in TCGA were downloaded from the UCSC Xena browser (GDC hub: https://gdc.xenahubs.net). No patients from the TCGA data set had any previous record of immunotherapy treatment. First, we selected patients with *NOTCH1*, *NOTCH2*, *NOTCH3*, *NOTCH4*, *NUMB*, *FBXW7*, *ADAM10*, *ADAM17* and γ‐secretase (*APH1A*, *APH1B*, *NCSTN*, *PSEN1* and *PSEN2*) mutations as the Notch mutation group, and germline mutations were excluded. Multiple somatic mutations including non‐synonymous mutations and insertion‐deletion mutations were all used for further analysis. We divided all mutations of each gene into overlap mutation and single‐mutation groups. If more than one kind of gene mutated, they were assigned to the overlap mutation group. Single mutation was defined as a mutation only in one kind of gene. Then, we analysed gene expression profiles and clinicopathologic characteristics between the two groups. The mRNA expression data, which were generated by the Illumina HiSeq V2 platform, were presented as RPKM and transformed into log2 values for further analysis. We calculated the average expression of immune checkpoint molecules, effector molecules as well as chemokines and chemokine receptors after z‐score normalization with log‐transformed expression profiles. Gene ontology (GO) analysis was performed to identify pathways that were up‐ and down‐regulated between the different groups using the R package cluster Profiler. We used gene set enrichment analysis (GSEA) (GSEA v4.0.1) to identify gene sets and pathways enriched in the mutation group and non‐mutation group. For calculating T cell subsets infiltration scores in the mutation and non‐mutation groups of the TCGA data set, we used single‐sample gene set enrichment analysis (ssGSEA) according to Yasin et al.^11^ Marker genes for immune cell types were from Yasin and Pornpimol et al^11^, ^12^ and are listed in supplementary materials. The *P* values were calculated by the Mann‐Whitney U test and used to determine the statistical significance in IBM SPSS statistics 21. For survival analysis, we used the Kaplan‐Meier method to generate survival curves, and the log‐rank test to determine the statistically significant differences in survival.

### 2.4 Other cancers from TCGA data sets analysis

To confirm whether Notch pathway mutation can have the similar effects on other malignant tumours, we downloaded data sets of 13 other types of cancers, including liver cancer, breast cancer, prostate cancer, kidney cancer, metastatic melanoma, non–small‐cell lung cancer (adenocarcinoma and squamous cancer), head and neck squamous cancer, gastric cancer, glioma, cervical cancer, ovarian cancer, bladder cancer and pancreatic cancer. The somatic mutation and mRNA expression data sets for these cancers were obtained from TCGA (http://www.cbioportal.org/datasets). Germline mutations were excluded. First, we selected patients with or without Notch signalling pathway mutations as described above for CRC. GO analysis was performed to identify pathways that were up‐ and down‐regulated between the two groups using the R package cluster Profiler.

### 2.5 Statistical analysis

R 3.5.3 was employed to analyse the GSE108989 data set and TCGA data sets. Difference plots of different groups were generated by GraphPad Prism 6. All *P* values were two‐sided, and *P* value of less than 0.05 was considered statistically significant.

## 3 RESULTS

### 3.1 Mutations in Notch signalling pathways are associated with increased tumour infiltration of cytotoxic CD8^+^ T cells in CRC patients

**Figure 1:**
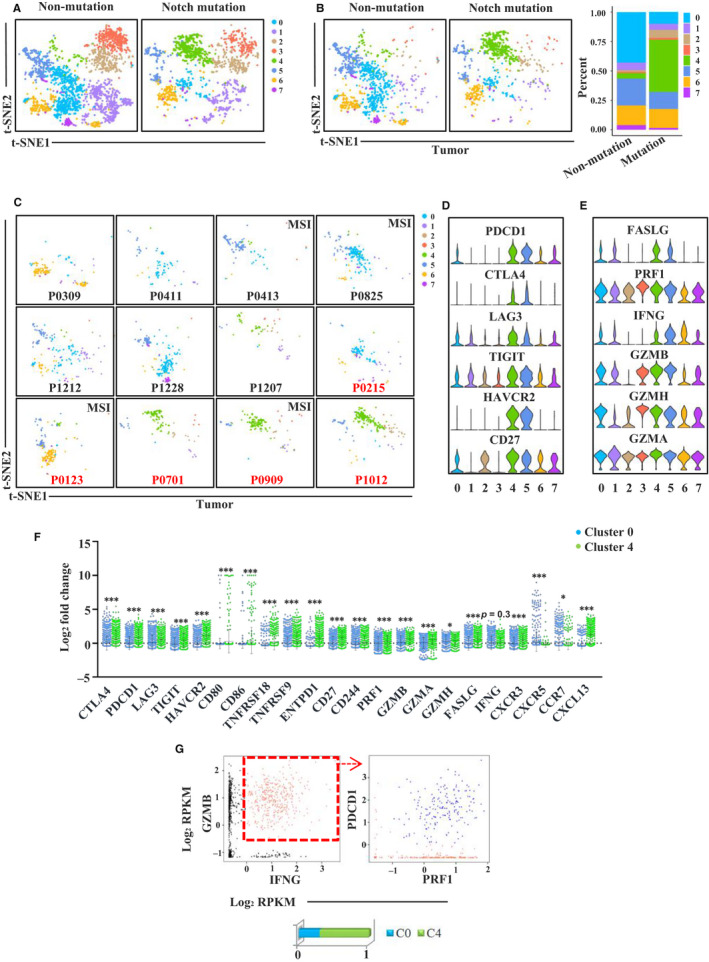
Gene expression profiles of CD8^+^ T cells in GSE108989 single T cell sequencing data set. A, t‐SNE plot of CD8^+^ T cells from Notch signalling pathway mutation and non‐mutation patients. B, t‐SNE and proportion plots of CD8^+^ T cells derived from tumour microenvironment between Notch signalling pathway mutation and non‐mutation patients. C, t‐SNE plot of CD8^+^ T cells derived from tumour microenvironment of each patient. Patients in red are Notch signalling pathway mutation ones. D, Violin plots showing normalized expression of immune checkpoint molecules in CD8^+^ T cell clusters. E, Violin plots showing normalized expression of cytotoxic related molecules in CD8^+^ T cell clusters. F, The expression of immune checkpoints, effector molecules and chemokines and chemokine receptors in cluster 0 and cluster 4 (*P < 0.05, ***P < 0.001, tested by Mann‐Whitney U test). G, Proportions of cells co‐expressing *GZMB, IFNG, PRF1* and *PDCD1* in clusters 0 and 4

To investigate the effect of Notch signalling pathway mutations on the CRC immune microenvironment, we analysed gene expression and whole exome sequencing data obtained from the GSE108989 data set. The different somatic mutations in the Notch signalling pathway of 12 CRC patients are shown in Table S1. P0701 had a stop‐gain mutation and a missense mutation in *FBXW7*. P1012 had a missense mutation in *NOTCH1*. P0215 had a stop‐gain mutation in *FBXW7*. P0123 had a missense mutation in both *NOTCH2* and *FBXW7*. P0909 had a frameshift deletion mutation in *FBXW7* and a missense mutation in *NOTCH1*. T‐SNE analysis identified eight clusters (0‐7) of CD8^+^ T cells in tumour, adjacent normal mucosa and peripheral blood. The top 20 genes of each cluster are listed in Table S2. Figure S1C and S1D shows the expression levels of the top 5 genes of each cluster (Figure S1C,D). We found that patients with Notch signalling pathway mutations possessed a cluster distribution profile different from that of patients without Notch pathway mutations (Figure 1A). Among the eight clusters, cluster 0 was enriched in the non‐mutation group, whereas cluster 4 was enriched in the Notch signalling pathway mutation group. To determine whether the differences were systemic or tumour microenvironment‐specific, we performed t‐SNE analysis of CD8^+^ T cells in different tissues (tumour, adjacent normal mucosa and peripheral blood). The distribution patterns of different clusters in adjacent normal mucosa and peripheral blood, however, were almost the same between the mutation group and the non‐mutation group (Figure S1A). Therefore, the different enrichment of clusters 0 and 4 in these two groups was specific to the tumour microenvironment (Figure 1B).

Most cells in cluster 4 were from the tumours of three patients (P0701, P1012 and P0909) with Notch signalling pathway mutations, whereas most cells in cluster 0 were from tumours of patients without mutations (Figure 1C). Interestingly, we found an enrichment of cluster 4 in tumour of P1207 without *NOTCH1*/*NOTCH2*/*FBXW7* mutations, but it is not known whether or not P1207 had mutations in other members of the Notch signalling pathway. CD8^+^ T cell from tumours of P0215 and P0123 did not show an enrichment of cluster 4 compared with the other three patients that had Notch signalling pathway mutations, indicating that different mutation types and mutated genes might have different consequences. Gene expression profile analysis in the eight clusters showed different gene expression levels of immune checkpoints and effector molecules (Figure 1D,E). We observed higher expression levels of immune checkpoint molecules (*CTLA4*, *PDCD1*, *LAG3*, *TIGIT*, *HAVCR2*, *TNFRSF9*, *TNFRSF18*, *CD27*, *CD80* and *CD86*), some effector molecules (*GZMB*, *PRF1*, *GZMA*, *FASLG* and *GZMH*), and also some chemokines and chemokine receptors (*CXCL13* and *CXCR3*) in cluster 4 compared to cluster 0 (Figure 1F). Because of the high expression of inhibitory molecules, cluster 4 tended to be exhausted. However, it has been proved that tumour‐specific CD8^+^ T cells are enriched among PD‐1 + cells, which also have a higher expression of TIM‐3 and LAG‐3.^13^, ^14^ According to the gene expression profiles, cluster 4 may be more tumour‐specific. We then identified cells with co‐expressions of *GZMB*, *IFNG*, *PRF1* and PDCD1, which were shown to have strong activity and cytotoxicity, and calculated the proportion of these cells in clusters 4 and 0 (Figure 1G). About 70% of these cells were found in cluster 4 and 30% in cluster 0 (Figure 1G), indicating that cluster 4 had stronger cell killing ability than cluster 0.

Furthermore, to investigate the influence of other mutations on immune response of CRC patients, we also analysed CD8^+^ T cells distribution patterns in patients with MSI, *BRAF*, *TP53* and *APC* mutations. However, similar distribution patterns and proportions of different CD8^+^ T cells clusters were observed between mutation group and non‐mutation group of these genes (Figure S2A‐D). These findings indicate that the Notch signalling pathway mutations are associated with enhanced CD8^+^ T cell anti‐tumour immunity in CRC.

### 3.2 Mutations in Notch signalling pathways are associated with impaired tumour infiltration of inhibitory Treg cells in CRC patients

**Figure 2:**
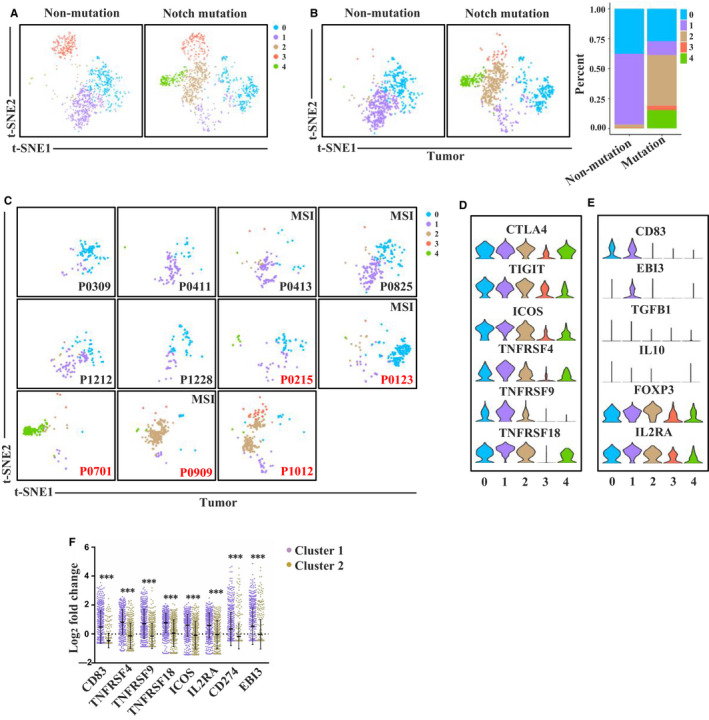
Gene expression profiles of Treg cells in GSE108989 single T cell sequencing data set. A, t‐SNE plot of Treg cells from Notch signalling pathway mutation and non‐mutation patients. B, t‐SNE plot of Treg cells derived from tumour microenvironment between Notch signalling pathway mutation and non‐mutation patients. C, t‐SNE plot of Treg cells derived from tumour microenvironment of each patient. Patients in red are Notch signalling pathway mutation ones. D, Violin plots showing normalized expression of immune checkpoints in Treg cell clusters. E, Violin plots showing normalized expression of inhibitory function related molecules in Treg cell clusters. F, The expression of immune checkpoint and inhibitory function related molecules in cluster 1 and cluster 2 (***P < 0.001, tested by Mann‐Whitney U test)

Tumour‐infiltrating Treg cells are critical for suppressing anti‐tumour immunity. To further study the effect of Notch signalling pathway mutations on the CRC immune microenvironment, we analysed the characteristics of Treg cells among these patients. T‐SNE analysis identified five Treg cell clusters (0‐4) from tumour, adjacent normal mucosa and peripheral blood. The top 20 genes of each cluster are listed in Table S3. We found that patients with Notch pathway mutations had different cluster profiles compared to patients without Notch pathway mutations (Figure 2A). Among the five clusters, cluster 1 was enriched in the non‐mutation group, whereas clusters 2 and 4 in the mutation group. We further performed t‐SNE analysis of Treg cells from different tissues (tumour, adjacent normal mucosa and peripheral blood). The distribution patterns of the different clusters in adjacent normal mucosa and peripheral blood were almost the same between the mutation group and the non‐mutation group (Figure S3A). In contrast, the distribution of Treg subsets in tumours was different between the two groups (Figure 2B). Most of the cells in cluster 1 were from tumours in the non‐mutation group. Most of the cells in cluster 2 were from tumours of P0909 (*NOTCH1* and *FBXW7* mutations) and P1012 (*NOTCH1* mutation), and most of the cells in cluster 4 were from tumour of P0701 (two types of *FBXW7* mutations) (Figure 2C). Gene expression profile analysis of the five clusters showed different gene expression levels of immune checkpoints (*CTLA4*, *TIGIT*, *ICOS*, *TNFRSF9*, *TNFRSF4* and *TNFRSF18*) as well as genes related to the inhibitory function of Treg cells (*CD83*, *EBI3*, *IL2RA* and *FOXP3*) (Figure 2D,E). Interestingly, cluster 1 Treg cells had exclusively high expression of *EBI3*, which encodes a critical subunit of IL‐35 (Figure 2E,F). Treg cell–derived IL‐35 has been reported to suppress anti‐tumour immunity and limit the differentiation of memory CD8^+^ T cells.^15^, ^16^ Additionally, the expression level of CD83 was also higher in cluster 1 than in clusters 2 and 4 (Figure 2E,F). Mice with Treg‐intrinsic CD83 deficiency are characterized by a pro‐inflammatory phenotype. Loss of CD83 in Treg cells has been shown to lead to the down‐regulation of Treg‐specific differentiation markers and induction of an inflammatory profile.^17^ These results indicate that cluster 1 is more inhibitory than clusters 2 and 4. Actually, cluster 1 Treg cells also expressed higher level of *FOXP3*, *IL2RA*, *ICOS*, *TNFRSF4*, *TNFRSF9* and *TNFRSF18*, indicating a stronger suppressive function than other Treg cells.

We also investigated Treg cell distribution patterns in patients with MSI, *BRAF* mutation, *TP53* mutation and *APC* mutation. However, similar distribution patterns and proportions of the different Treg cells clusters were observed between the mutation and non‐mutation group of these genes (Figure S4A‐D). The t‐SNE analysis of conventional CD4^+^ T cells, however, showed fewer distribution differences than those of CD8^+^ T cells and Treg cells between the two groups (Figure S5). These results indicate that Notch signalling pathway mutations are associated with decreased *EBI3* and *CD83*‐expressing Treg cells in CRC tumour microenvironment, thus enhancing anti‐tumour immunity.

### 3.3 Notch signalling pathway mutations are associated with skewed immune landscape towards tumour suppression

**Figure 3:**
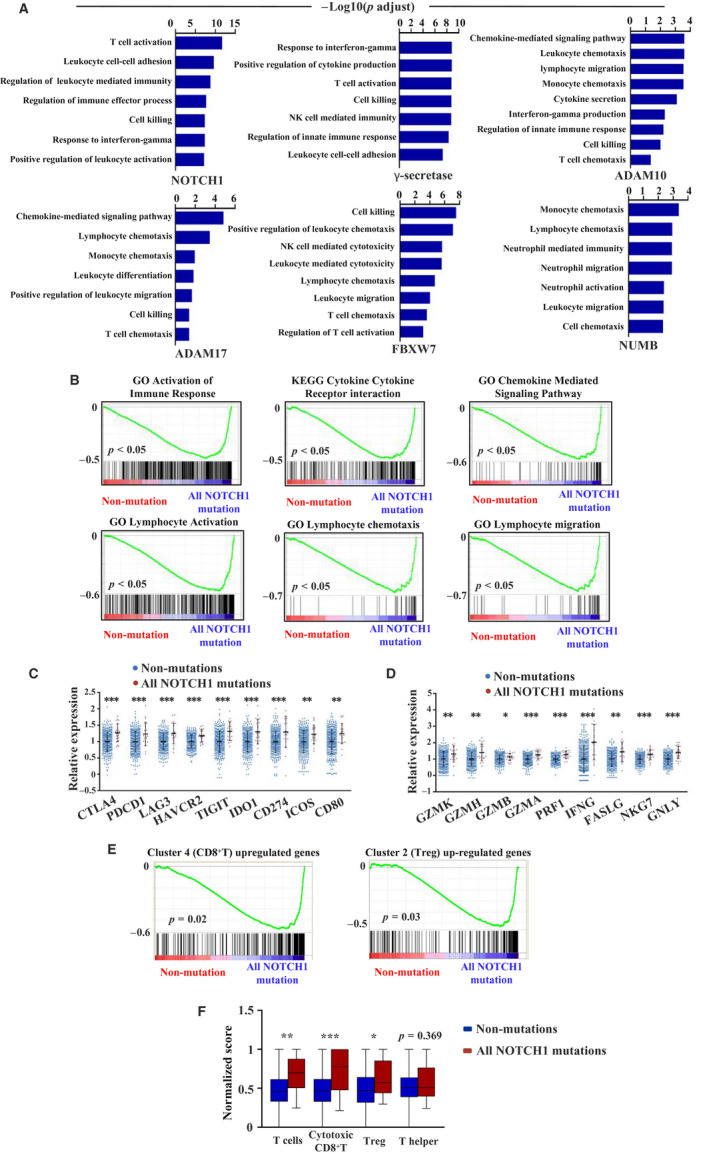
Gene set enrichment analysis of Notch pathway mutation (overlap mutation and single mutation) in TCGA data set. A, Up‐regulated gene sets in Notch signalling pathway mutation group (*NOTCH1*, γ‐secretase, *ADAM10, ADAM17, FBXW7* and *NUMB*) compared with non‐mutation group by GO analysis. B, Significant enrichment of immune‐related pathway between all *NOTCH1* mutation group and non‐mutation group by KEGG and GO analysis. C, Relative expression of immune checkpoint molecules in all *NOTCH1* mutation and non‐mutation groups (**P < 0.01, ***P < 0.001, tested by Mann‐Whitney U test). D, Relative expression of effector molecules in all *NOTCH1* mutation and non‐mutation groups (*P < 0.05, **P < 0.01, ***P < 0.001, tested by Mann‐Whitney U test). E, GSEA analysis of all *NOTCH1* mutation and non‐mutation groups. F, Normalized scores of different T cell subsets between all *NOTCH1* mutation group and non‐mutation group (*P < 0.05, **P < 0.01, ***P < 0.001, tested by Mann‐Whitney U test)

To validate the effect of Notch signalling pathway mutations on immune response observed in the GSE108989 data set, we used another CRC data set from the TCGA database to further analyse the relationship between Notch signalling pathway mutations and gene expression profiles. To investigate the effects of other important Notch signalling pathway‐related genes besides *NOTCH1*, *NOTCH2* and *FBXW7* found in GSE108989, we identified patients with somatic Notch signalling pathway mutations as described in Method. Table S4 shows the somatic mutations of Notch signalling pathway–related genes in the TCGA data set. Then, one by one, we separately analysed gene expression differences between the mutation group with *NOTCH1*, *NOTCH2*, *NOTCH3*, *NOTCH4*, *FBXW7*, *NUMB*, *ADAM10*, *ADAM17* and γ‐secretase mutations and the non‐mutation group. GO pathway analysis revealed that immune reaction processes were up‐regulated in the mutation groups (Figure 3A and Figure S6A). GSEA showed enrichment of genes associated with activation of immune responses in the mutation groups (Figure 3B). Because *NOTCH1* is the most important receptor in this pathway according to the results of the GSE108989 data set analysis, we evaluated gene expression differences between the all *NOTCH1* mutation group (including both overlap mutation and single mutation) and the non‐mutation group. We found significant differences in the expression levels of genes encoding immune checkpoint molecules (*CTLA4*, *PDCD1*, *LAG3*, *HAVCR2*, *TIGIT*, *IDO1*, *CD274*, *ICOS* and *CD80*) and effector molecules (*GZMK*, *GZMH*, *GZMB*, *GZMA*, *PRF1*, *IFNG*, *FASLG*, *NKG7* and *GNLY*) as well as chemokines (*CXCL9*, *CXCL10*, *CXCL11*, *CXCL13*, *CCL5* and *CCL18*) and chemokine receptors (*CXCR5*, *CXCR6* and *CCR5*) between the all *NOTCH1* mutation and non‐mutation groups (Figure 3C,D and Figure S6B).

Furthermore, we selected the up‐regulated genes in cluster 4 (CD8) and cluster 2 (Treg) and performed GSEA analysis on the all *NOTCH1* mutation and non‐mutation groups. The up‐regulated genes in the two clusters were significantly enriched in the all *NOTCH1* mutation group compared to the non‐mutation group (Figure 3E). To determine whether the infiltration of T cell subsets in all *NOTCH1* mutation group differs from that in the non‐mutation group, we calculated the infiltration scores of the two groups (Figure 3F). The scores of T, cytotoxic CD8^+^ T and Treg cells were higher in the all *NOTCH1* mutation group (Figure 3F). These results indicate that CD8^+^ T cells in CRC patients with Notch signalling pathway mutations are more cytotoxic, which is consistent with the results of GSE108989 data set. However, we did not observe any significant enrichment of the Notch signalling pathway between the mutation and non‐mutation groups (Figure S7). These results demonstrate that the Notch signalling pathway mutations can activate anti‐tumour immune responses in CRC patients.

### 3.4 Increased enrichment of immune response associated genes in CRC patients with overlap mutation than those with single mutation

**Figure 4:**
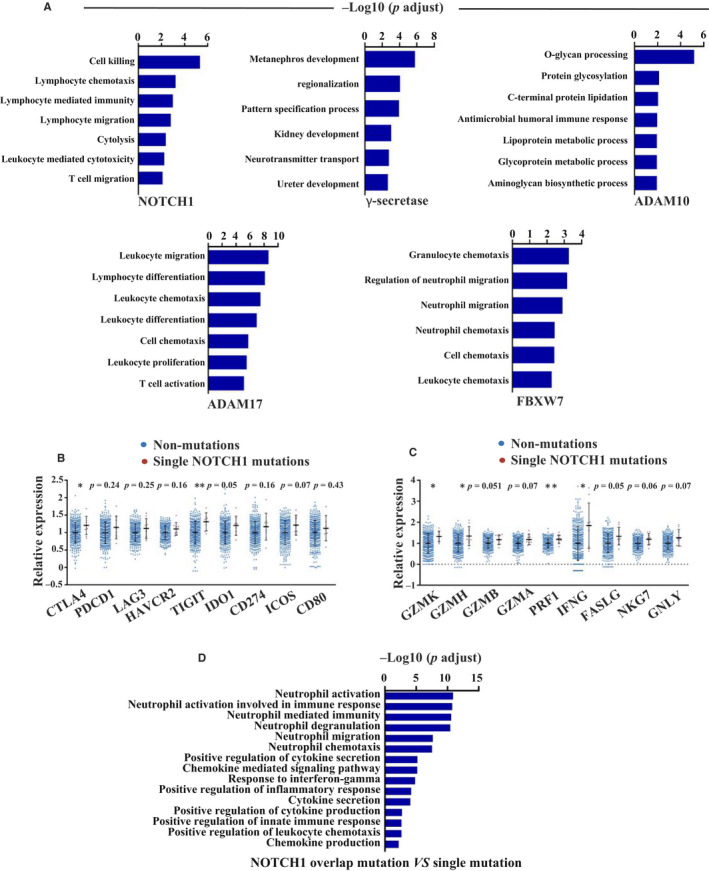
Gene set enrichment analysis of Notch pathway mutation (single mutation) in TCGA cohort. A, Up‐regulated gene sets in Notch signalling pathway single‐mutation group (*NOTCH1*, γ‐secretase, *ADAM10, ADAM17*, and *FBXW7*) compared with non‐mutation group by GO analysis. B, Relative expression of immune checkpoint molecules in single *NOTCH1* mutation and non‐mutation groups (*P < 0.05, **P < 0.01, tested by Mann‐Whitney U test). C, Relative expression of effector molecules in single *NOTCH1* mutation and non‐mutation groups (*P < 0.05, **P < 0.01, tested by Mann‐Whitney U test). D, Up‐regulated gene sets in *NOTCH1* overlap mutation compared with single mutation by GO analysis

According to the results obtained from the analysis of the GSE108989 data set, CD8^+^ T cells from P0909 with a frameshift deletion mutation in *FBXW7* and a missense mutation in *NOTCH1* showed different cluster distributions from those of P0215 with a stop‐gain mutation in *FBXW7*. Therefore, we hypothesized that an overlap mutation might induce a stronger immune response than a single mutation, so we compared the gene expression signatures of tumours between the single mutation in *NOTCH1*, *NOTCH2*, *NOTCH3*, *NOTCH4*, *FBXW7*, *ADAM10*, *ADAM17* and γ‐secretase and the non‐mutation groups as well as between the overlap *NOTCH1* mutation and single *NOTCH1* mutation groups. We excluded *NUMB* mutation because only one patient had a single *NUMB* mutation (Table S3). According to the GO analysis results, the number of enriched immune response processes in the single‐mutation group decreased compared with the all mutations group (Figure 4A and Figure S6A). Still, we found significant differences between the expression levels of some genes that were listed in Result 3 between the *NOTCH1* single‐mutation group and the non‐mutation group. The expression levels of effector molecules (*GZMB*, *GZMA*, *FASLG*, *NKG7* and *GNLY*), immune checkpoint molecules (*PDCD1*, *LAG3*, *HAVCR2*, *IDO1*, *CD274*, *ICOS* and *CD80*), and chemokines (*CXCL9*, *CCL5* and *CCL18*) and chemokine receptors (*CXCR6*), however, did not show any significant differences between the *NOTCH1* single‐mutation group and the non‐mutation group (Figure 4B,C and Figure S6C). Furthermore, genes involved in immune activation were enriched in the *NOTCH1* overlap‐mutation group compared with the *NOTCH1* single‐mutation group (Figure 4D). Altogether, these results indicate that overlap mutations in the Notch signalling pathway have a stronger effect on activating anti‐tumour immune responses than single mutations.

### 3.5 Notch signalling pathway mutations are associated with disease stage and metastasis, but not patient survival

To further investigate whether Notch signalling pathway mutations affect clinicopathological characteristics and the prognosis of patients with CRC, we analysed the clinicopathological characteristics and survival data between the mutation group (including *NOTCH1*, *NOTCH2*, *NOTCH3*, *NOTCH4*, *FBXW7*, *NUMB*, *ADAM10*, *ADAM17* and γ‐secretase mutations) and the non‐mutation group. We did not find any significant differences regarding age, sex, T stage and location (colon vs rectum) between the two groups. However, 47.4% patients in the non‐mutation group presented at advanced stages (stage III and stage IV), whereas 33.1% patients in the mutation group presented at advanced stages (stage III and stage IV) (*P* = 0.005). Compared to patients with mutations, individuals without mutations showed significantly higher rates of lymph node metastases and distant metastases (*P* = 0.013 and *P* = 0.035, respectively). The mutation group, however, had more mucinous cancers than the non‐mutation group (18.7% vs 10.9%, *P* = 0.016). The overall survival and disease‐free survival between the two groups showed no significant differences (*P* = 0.781 and *P* = 0.668) (Figure S8). Because the Notch signalling pathway mutations did not affect survival, these results suggest that the Notch signalling pathway mutation group, which had a greater proportion of patients with early stages of CRC, might respond differently to treatment compared with the non‐mutation group.

### 3.6 The effects of Notch signalling pathway mutation on tumour immune microenvironment vary in different cancers

**Figure 5:**
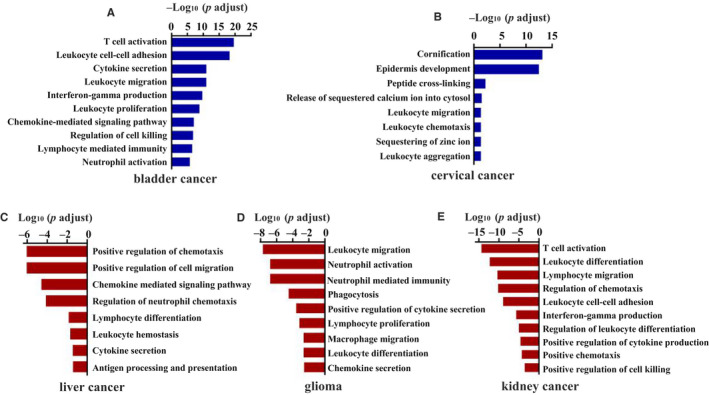
Gene set enrichment analysis of Notch signalling pathway mutation in other cancers from TCGA data sets. A, Bladder cancer. B, Cervical cancer. C, Liver cancer. D, Glioma. E, Kidney cancer

To determine whether Notch signalling pathway mutations can affect tumour immune responses in other cancers, we performed gene expression signature analysis in 13 other cancers. According to the gene enrichment analysis, leucocyte activation, adhesion and cytokine secretion pathways were up‐regulated in the mutation group with bladder cancer (Figure 5A), just as in CRC patients. Gene sets involved in leucocyte migration and chemotaxis were up‐regulated in patients with cervical cancer with mutations (Figure 5B). On the contrary, pathways involved in T cell activation, chemokine‐mediated signalling, and other pathways related to the activation of immune response were down‐regulated in patients with liver cancer, glioma and kidney cancer with Notch signalling pathway mutations (Figure 5C,D,E). In other cancers, however, the Notch signalling pathway mutations seemed to have little effect on the anti‐tumour immune response (Figure S9). These results indicate that the effects of Notch signalling pathway mutations on the tumour immune microenvironment depend on the tumour context.

## 4 DISCUSSION

Substantial evidence has indicated that the Notch signalling pathway plays an important role in tumorigenesis and development of CRC.^18^, ^19^, ^20^, ^21^, ^22^, ^23^, ^24^ Mutations of genes in the Notch signalling pathway have been reported in a number of human malignancies, in both haematopoietic and solid tumours.^25^ In this study, we first analysed the GSE108989 data set to investigate the effects of Notch signalling pathway mutations on the immunophenotype of CRC, which differs from the aim of the original article by Zhang et al^10^ to quantitatively analyse the dynamic relationships among 20 identified T cell subsets. The analysis of the GSE108989 data set indicated that gene mutations in the Notch signalling pathway could enhance CD8^+^ T cells anti‐tumour immunity and impair the inhibitory effect of Treg cells. In the TCGA data set, the immune activation pathways were highly up‐regulated in the mutation group compared with the non‐mutation group as expected. These findings suggest that Notch pathway mutations may activate the anti‐tumour immune responses of the CRC tumour microenvironment.

In the analysis of the GSE108989 data set, cluster 4 of CD8^+^ T cells can concomitantly express inhibitory and stimulatory molecules. In Zhang et al's original article,^10^ they defined these cells as exhausted T cells. However, they thought these cells still had anti‐tumour effector potential in vivo. Several studies have proved that tumour‐specific CD8^+^ T cells are enriched among PD‐1 + cells, which also show higher expression of TIM‐3 and LAG‐3.^13^, ^14^ These results indicate that cluster 4 tends to be exhausted and tumour‐specific subset, as cluster 4 also expresses high levels of effector molecules such as *FASLG*, *PRF1*, *GZMB*, *GZMH* and *GZMA*, which may indicate a strong ability to kill cancer cells.

According to the GSE108989 data set, not all Notch signalling–related gene mutations induce CD8^+^ T cell activation. P0123 (with a missense mutation in *NOTCH2* and *FBXW7*) showed different CD8^+^ T cell cluster distributions from P0909 (with a frameshift deletion mutation in *FBXW7* and a missense mutation in *NOTCH1*), which indicated that Notch1 may be the most important of the four Notch receptors. P0215, with a stop‐gain mutation in *FBXW7,* seemed to have no effect on anti‐tumour immune responses, unlike P0701 with a stop‐gain mutation and a missense mutation of *FBXW7*, suggesting that different mutation types and sites of Notch signalling‐related genes might have different effects on anti‐tumour immune response.

It has been reported that the dysregulation of the Notch pathway in the tumour skews the local cytokine milieu, shaping the immunological landscape and affecting tumour growth, progression and metastases.^26^ The Notch signalling pathway is a key regulator of inflammatory‐related cytokines in cancers, such as IL‐10,^27^ TGF‐β,^28^ IL‐6,^29^ CCL2^30^ and CXCL12.^31^ In our study, we observed that the expression of *CXCR3* was up‐regulated in cluster 4 (mainly from tumours of patients with Notch pathway mutations) compared with cluster 0 (mainly from tumours of non‐mutation patients) of CD8^+^ T cells. Moreover, the ligands of CXCR3 (*CXCL9*, *CXCL10* and *CXCL11*) were higher in the mutation group in the TCGA data set. CXCR3 and its ligands played essential roles in the spatial distribution, migratory behaviour and function of T cells.^32^ They can guide the recruitment of effector T cells into the inflamed peripheral tissue.^33^ Engineering tumour cells with expression of CXCL10 can induce an anti‐tumour immune response.^34^ CXCR3 expression on CD8^+^ T cells is critical for their entry into tumours^35^ and is required for the enhancement of the intratumoural CD8^+^ T cell response under the treatment of PD‐1 blockade.^36^ CXCR3 ligand expression might serve as early biomarkers of response to checkpoint blockade therapy in melanoma patients.^36^ These results suggest that CXCR3 and its ligands interaction might be a potential pathway to mediate the effect of Notch mutations on the tumour immune microenvironment. The mechanisms, however, need further investigation. In addition, CRC patients with Notch pathway mutations may be a promising biomarker for immune checkpoint blockade therapy.

The influences of Notch signalling alterations are always context‐dependent. Aberrantly activated Notch signalling has been observed during the carcinogenesis of many human cancers in addition to CRC, including pancreatic cancer,^37^ breast cancer,^38^ prostate cancer,^39^ liver cancer,^40^ cervical cancer,^41^ lung cancer,^42^ ovarian cancer^43^ and renal cancer.^44^ The effects of Notch pathway mutations on patients with bladder cancer and cervical cancer were almost the same as those for patients with CRC. However, contradictory effects were observed in patients with kidney cancer, glioma and liver cancer with Notch pathway mutations. In summary, the functional roles of Notch pathway mutations in different types of cancer are highly context‐dependent. Further studies are needed to explore the context‐specific interaction between Notch signalling mutations in cancer cells and the anti‐tumour immune response.

This study revealed that Notch pathway mutations in CRC patients can activate anti‐tumour immune responses with checkpoint molecules up‐regulation, which indicated that this kind of patients with CRC may be sensitive to immune checkpoint antibody therapy. This finding may provide precise treatment strategies for CRC patients with Notch pathway mutation.

This study, however, had several drawbacks. Because of the limited sample size of the TCGA data set, it was hard to assess the influences of different mutation types (insertion‐deletion and point mutation) and mutation sites on tumour immune response. Furthermore, only limited information of whole exome sequencing data could be obtained from the GSE108989 data set. Thus, we had no idea whether this data set had other Notch pathway‐related gene mutations. Precise and deeper understandings of Notch mutation effects on the tumour microenvironment are expected to elucidate the mechanisms of immune response activation.

## CONFLICT OF INTEREST

The authors confirm that there are no conflicts of interest.

## AUTHOR CONTRIBUTION

**Fei Wang:** Data curation (lead); Formal analysis (lead); Investigation (lead); Writing‐original draft (lead). **Jie Long:** Data curation (equal); Formal analysis (equal); Software (lead); Writing‐original draft (equal). **Liang Li:** Formal analysis (equal); Validation (equal). **Zhi‐bin Zhao:** Methodology (equal); Writing‐review & editing (equal). **Fang Wei:** Data curation (equal); Investigation (equal). **Yuan Yao:** Methodology (equal); Writing‐review & editing (supporting). **Wenjing Qiu:** Data curation (supporting); Formal analysis (supporting). **Zixin Wu:** Data curation (supporting); Formal analysis (supporting). **Qingqing Luo:** Data curation (supporting); Formal analysis (supporting). **Wei Liu:** Methodology (supporting); Writing‐review & editing (supporting). **Yibo Quan:** Methodology (supporting); Writing‐review & editing (supporting). **Zhexiong Lian:** Funding acquisition (equal); Supervision (equal); Writing‐review & editing (equal). **Jie Cao:** Funding acquisition (lead); Supervision (lead); Writing‐review & editing (lead).

## Supporting information

Supplementary Material

Fig S1‐S9

Table S1‐S4

## Data Availability

The single T cell sequencing raw data are available as GEO accession GSE108989: https://www.ncbi.nlm.nih.gov/geo/query/acc.cgi?acc = GSE108989. Gene expression data, whole exome sequencing data and clinicopathological data of TCGA CRC data set were downloaded from the UCSC Xena browser (GDC hub: https://gdc.xenahubs.net).
