# Supplementary material for: Mutations in the notch signalling pathway are associated with enhanced anti‐tumour immunity in colorectal cancer
Source: J Cell Mol Med. 2020 Sep 14;24(20):12176–87. doi: 10.1111/jcmm.15867 (PMC7579712; doi:10.1111/jcmm.15867)
Supplement: Supplementary file 2 — Fig S1‐S9 [file JCMM-24-12176-s002.pdf]

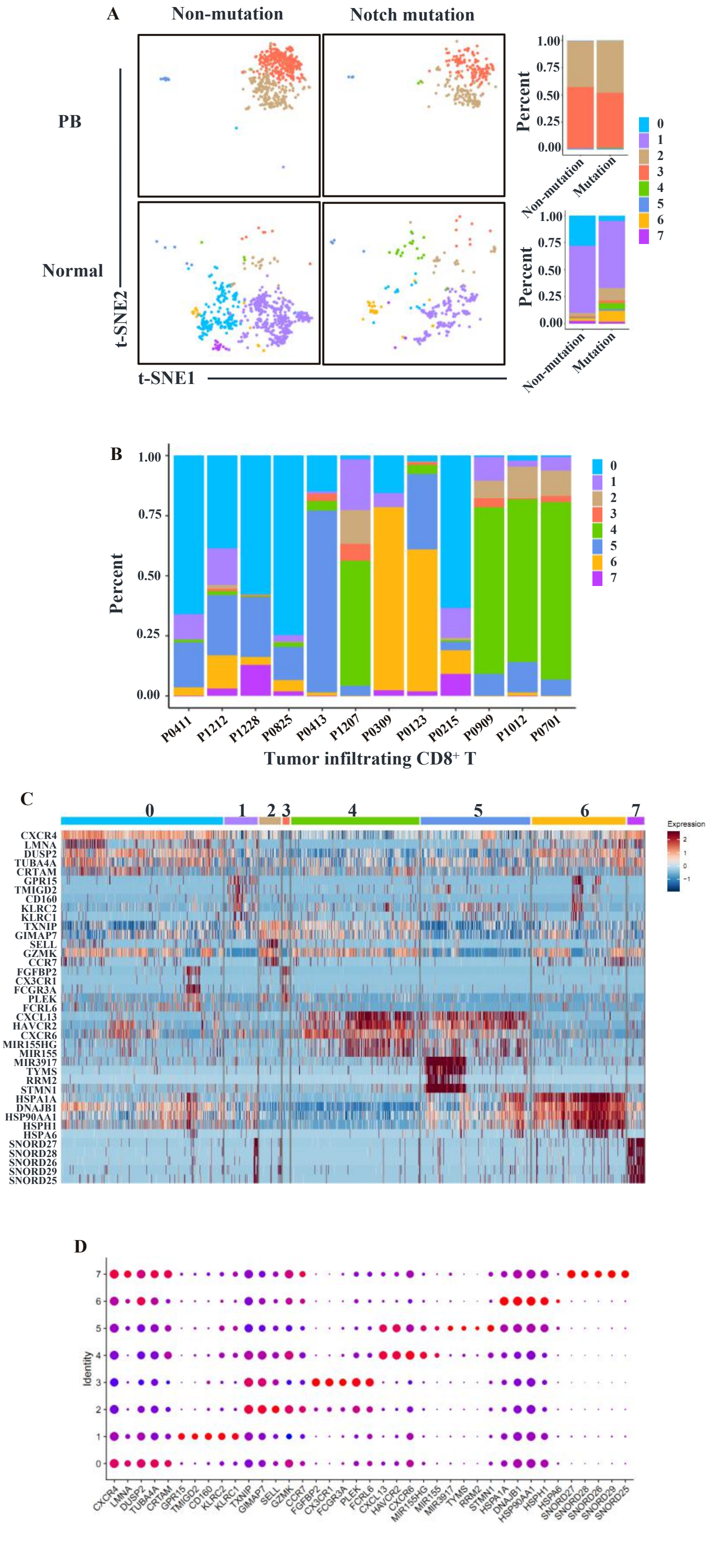

Figure S1 Gene expression profiles of CD8+ T cells in GSE108989 single T cell sequencing dataset.
A, t-SNE and proportion plots of CD8+ T cells derived from peripheral blood and adjacent normal mucosa between Notch pathway mutation and non-mutation patients. B, proportion plot of eight CD8+ T cell clusters derived from tumor microenvironment in twelve patients. C, heat map showing the top 5 genes expression levels of eight CD8+ T cell clusters. D, relative gene expression levels of eight CD8+ T cell clusters.

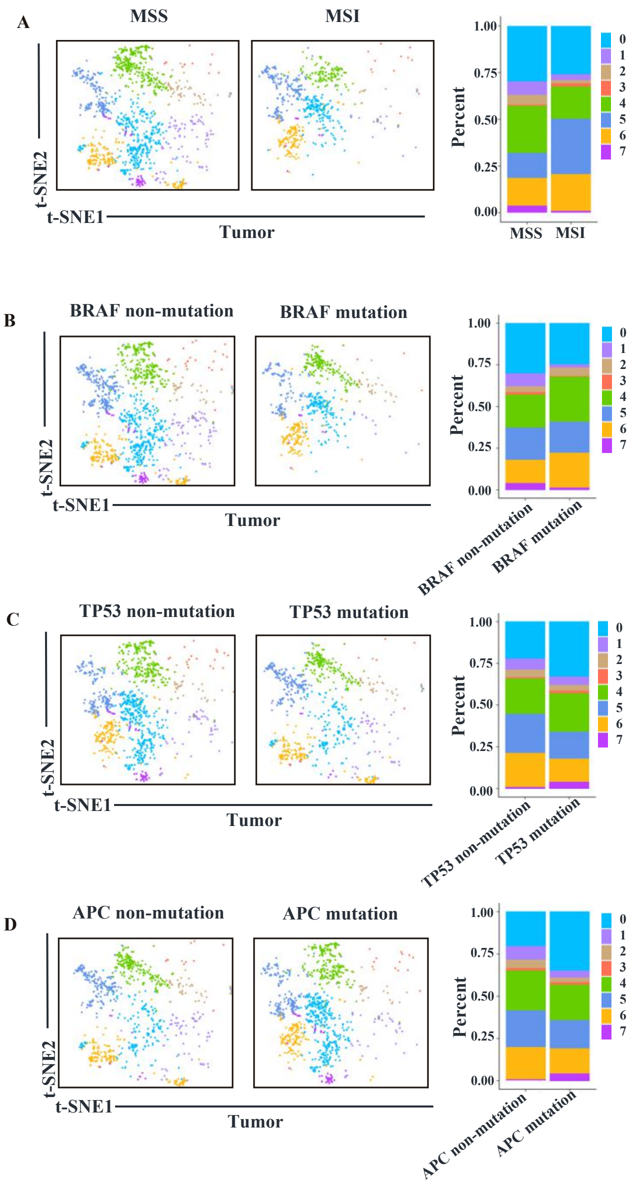

Figure S2 Gene expression profiles of CD8^{+} T cells in patients with different mutations. A, t-SNE and proportion plots of CD8^{+} T cells derived from tumor microenvironment between MSS and MSI patients. B, t-SNE and proportion plots of CD8^{+} T cells derived from tumor microenvironment between BRAF non-mutation and mutation patients. C, t-SNE and proportion plots of CD8^{+} T cells derived from tumor microenvironment between TP53 non-mutation and mutation patients. D, t-SNE and proportion plots of CD8^{+} T cells derived from tumor microenvironment between APC non-mutation and mutation patients.

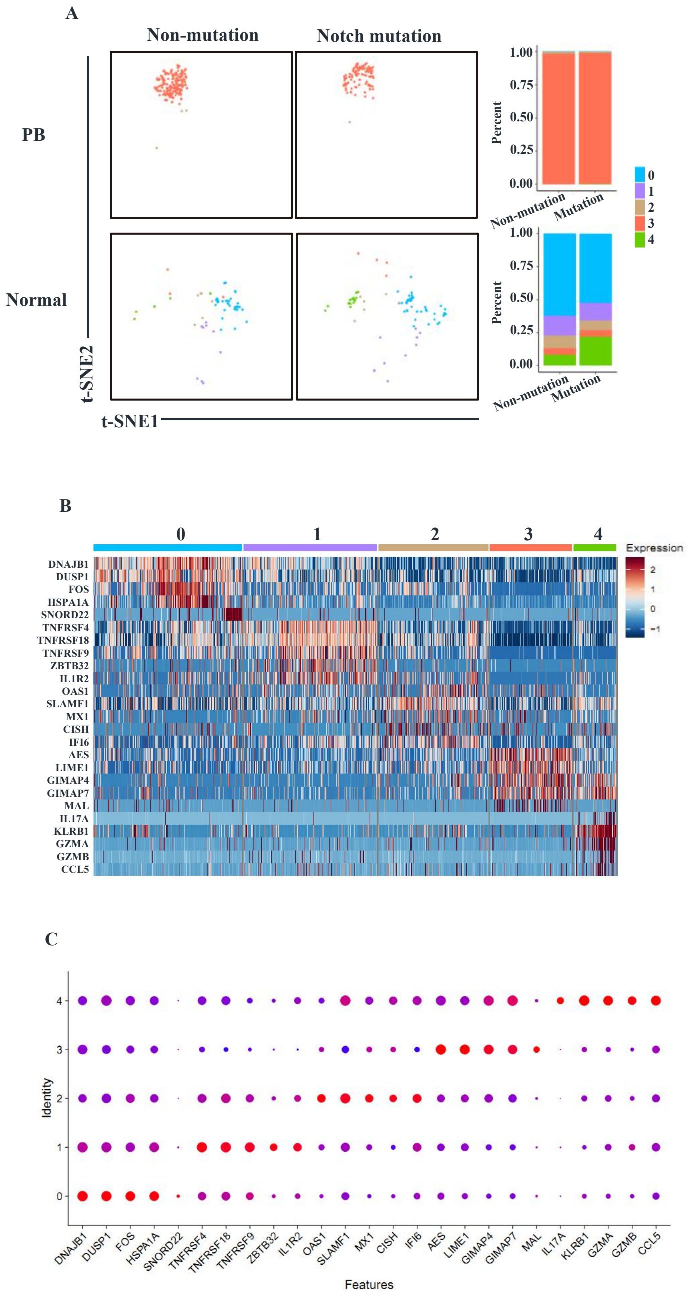

Figure S3 Gene expression profiles of Treg cells in GSE108989 single cell sequencing dataset.

A, t-SNE and proportion plots of Treg cells derived from peripheral blood and adjacent normal mucosa between Notch pathway mutation and non-mutation patients. B, heat map showing the top 5 genes expression levels of five Treg cell clusters. C, relative gene expression levels of five Treg cell clusters.

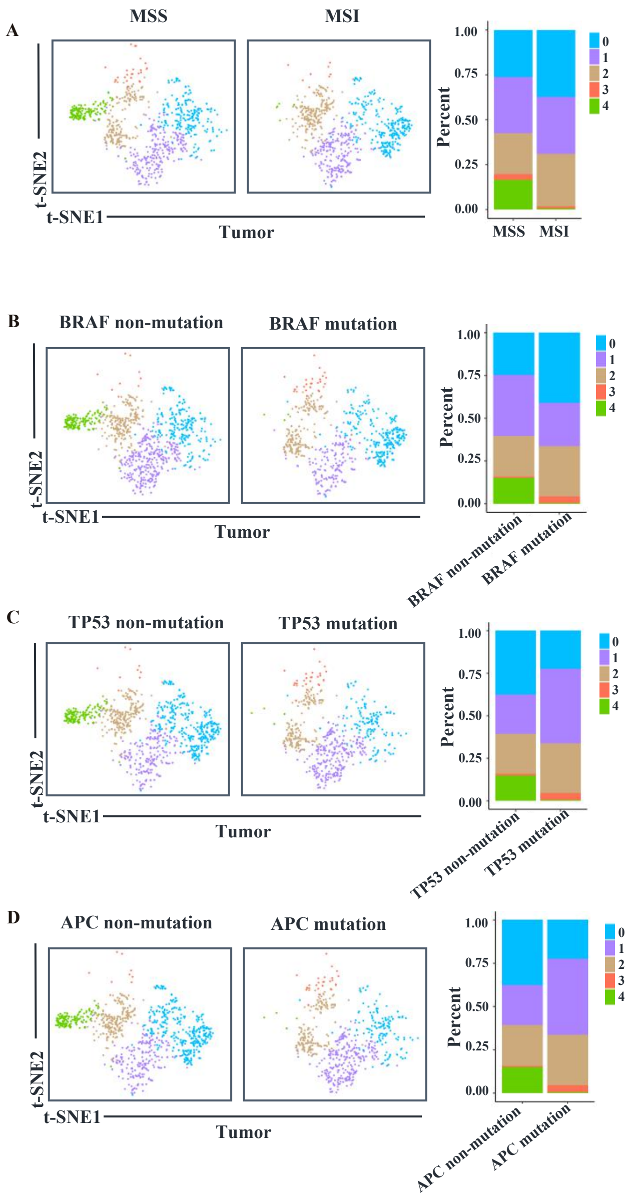

Figure S4 Gene expression profiles of Treg cells in patients with different mutations. A, t-SNE and proportion plots of Treg cells derived from tumor microenvironment between MSS and MSI patients. B, t-SNE and proportion plots of Treg cells derived from tumor microenvironment between BRAF non-mutation and mutation patients. C, t-SNE and proportion plots of Treg cells derived from tumor microenvironment between TP53 non-mutation and mutation patients. D, t-SNE and proportion plots of Treg cells derived from tumor microenvironment between APC non-mutation and mutation patients.

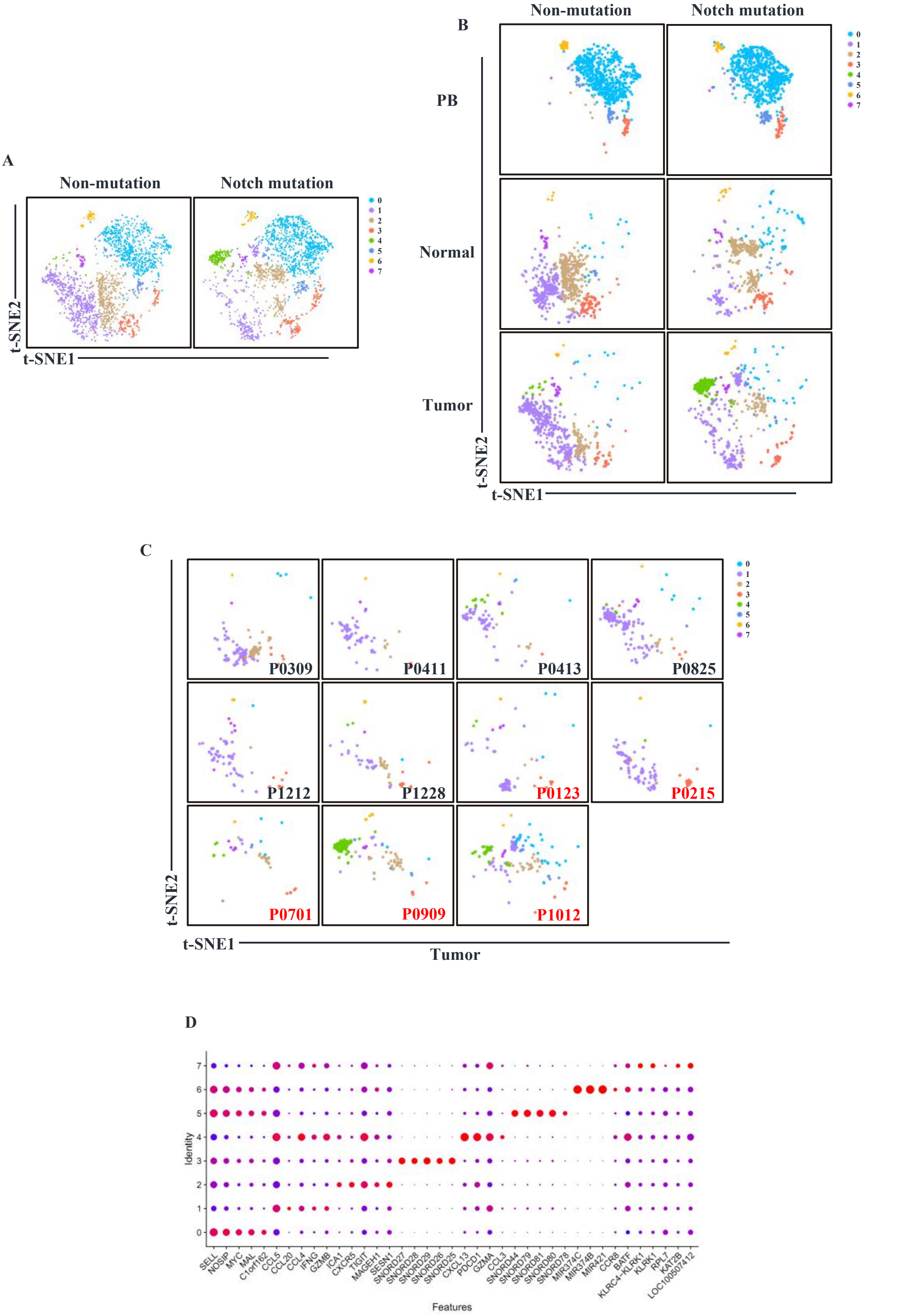

Figure S5 Gene expression profiles of conventional CD4⁺ T cells in GSE108989 single cell sequencing dataset.

A, t-SNE plot of conventional CD4⁺ T cells from Notch pathway mutation and non-mutation patients. B, t-SNE plot of conventional CD4⁺ T cells derived from peripheral blood, adjacent normal mucosa and tumor between Notch pathway mutation and non-mutation patients. C, t-SNE plot of conventional CD4⁺ T cells derived from tumor microenvironment of each patient. D, relative gene expression levels of eight conventional CD4⁺ T cell clusters.

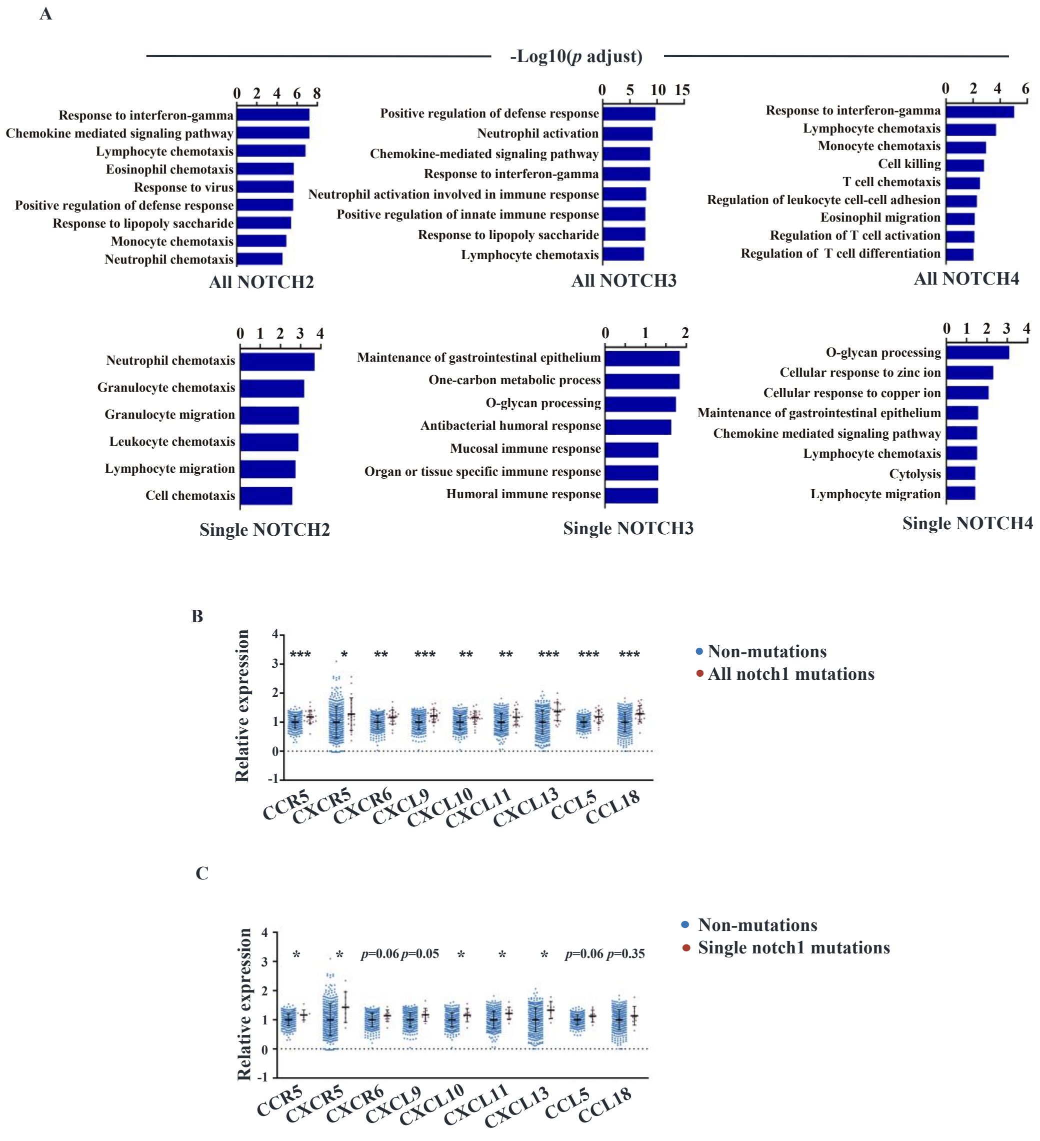

Figure S6 Gene set enrichment analysis of Notch signaling pathway in TCGA dataset.
A, up regulated gene sets in Notch signaling pathway mutation group (NOTCH2, NOTCH3, NOTCH4) compared with non-mutation group by GO analysis. B, relative expression of chemokines and chemokine receptors in all NOTCH1 mutation and non-mutation groups (*p<0.05, **p<0.01, ***p<0.001, tested by Mann-Whitney U test). C, relative expression of chemokines and chemokine receptors in single NOTCH1 mutation and non-mutation groups (*p<0.05,tested by Mann-Whitney U test).

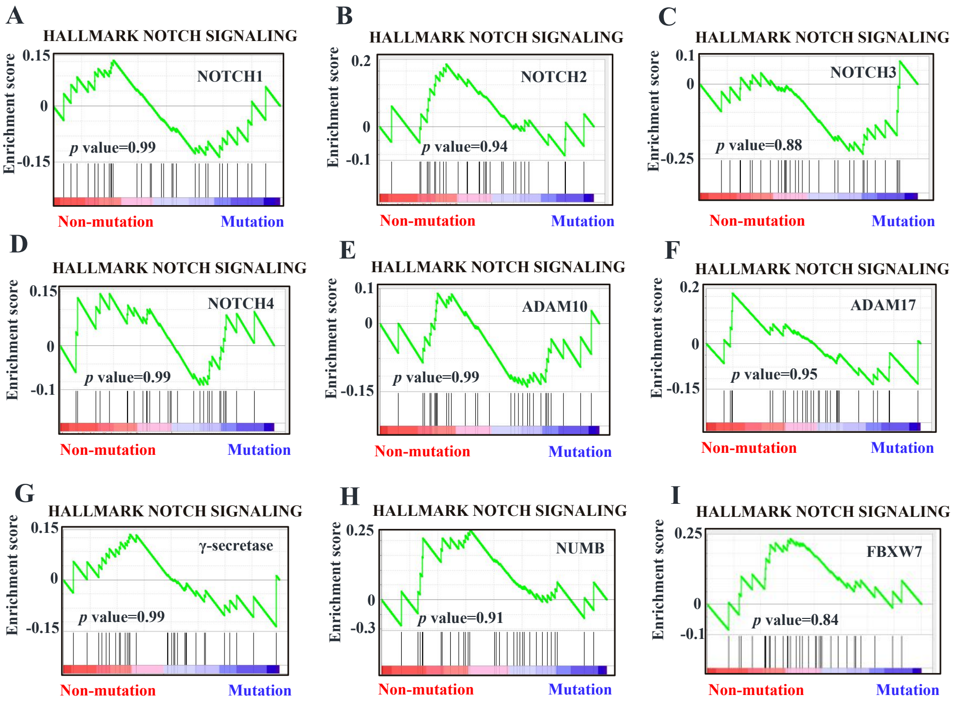

Figure S7 Notch signaling pathway enrichment analysis between mutation and non-mutation groups in TCGA dataset.
A, Notch pathway enrichment analysis between all NOTCH1 mutation and non-mutation group by HALLMARK. B, NOTCH2. C, NOTCH3. D, NOTCH4. E, ADAM10. F, ADAM17. G, γ-secretase. H, NUMB. I, FBXW7.

A

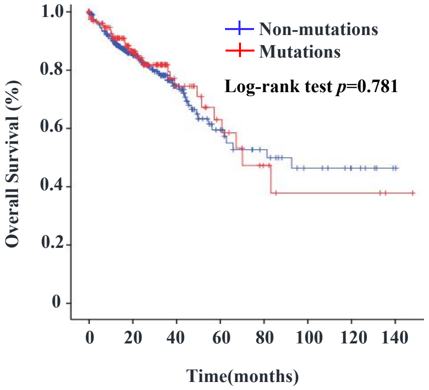

B

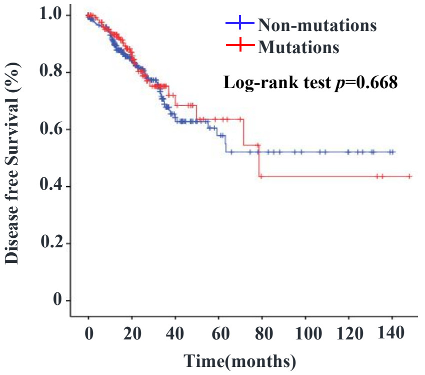

Figure S8 Prognostic analysis between mutation and non-mutation groups in TCGA dataset. A, Overall survival; B, Disease free survival.

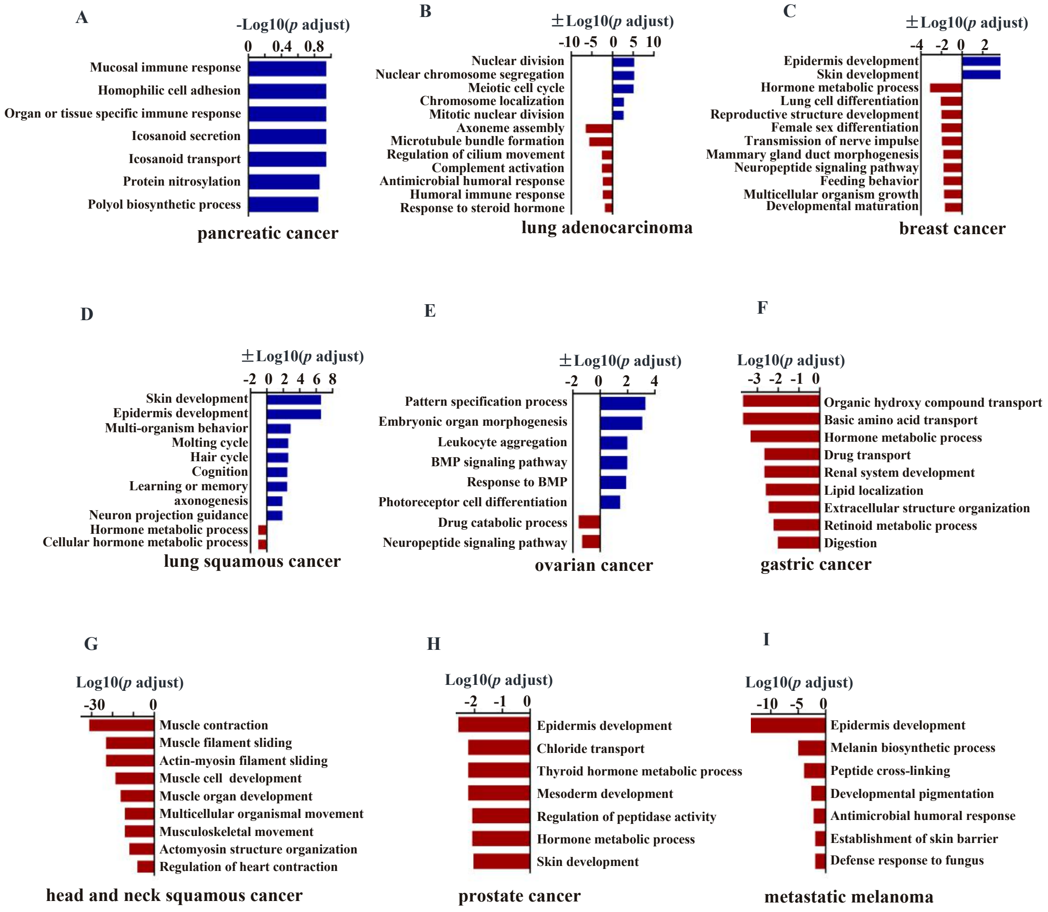

Figure S9 Gene set enrichment analysis of Notch signaling pathway mutation in other cancers from TCGA datasets.

A, pancreatic cancer. B, lung adenocarcinoma. C, breast cancer. D, lung squamous cancer. E, ovarian cancer. F, gastric cancer. G, head and neck squamous cancer. H, prostate cancer. I, metastatic melanoma. Pathways in blue are up-regulated gene sets in Notch signaling pathway mutation group, and these in red are down-regulated in mutation group.
