# Supplementary material for: Mutations in the notch signalling pathway are associated with enhanced anti‐tumour immunity in colorectal cancer
Source: J Cell Mol Med. 2020 Sep 14;24(20):12176–87. doi: 10.1111/jcmm.15867 (PMC7579712; doi:10.1111/jcmm.15867)
Supplement: Supplementary file 3 — Table S1‐S4 [file JCMM-24-12176-s003.pdf]

Table 1 Clinicopathological characteristics between mutation and non-mutation groups

	Non-mutations(368)	Mutations(157)	*p*
Age(years)	65.41±12.415	66.87±14.264	0.267
Sex			0.653
male	192	79	
female	174	78	
Stage			0.005
I	66(18.8)	27(17.5)	
II	119(33.8)	76(49.4)	
III	112(31.8)	38(24.7)	
IV	55(15.6)	13(8.4)	
T			0.61
Tis	1(0.3)	0(0)	
T1	12(3.3)	5(3.2)	
T2	69(18.9)	25(15.9)	
T3	241(65.8)	113(72)	
T4	43(11.7)	14(8.9)	
N			0.013
N0	198(54.2)	107(68.2)	
N1	98(26.8)	30(19.1)	
N2	69(18.9)	20(12.7)	
M			0.035
M0	263(83)	125(90.6)	
M1	54(17)	13(9.4)	
Location			0.79
colon	267(74.4)	117(75.5)	
rectum	92(25.6)	38(24.5)	
Pathology			0.016
adenocarcinoma	320(89.1)	126(81.3)	
mucinous	39(10.9)	29(18.7)	

Table S1 Somatic mutations of notch pathway detected by whole exome sequencing in single cell sequencing cohort

Patient	Genomic mutation	Exonic function	Gene	c_DNA mutation	Protein mutation
P0701	4:153244185,G>A	stopgain	FBXW7	c.C1444T	p.R482X
	4:153247289,G>A	missense_SNV	FBXW7	c.C985T	p.R329C
P1012	9:139402561,C>G	missense_SNV	NOTCH1	c.G1046C	p.G349A
P0215	4:153332832,G>A	stopgain	FBXW7	c.C124T	p.Q42X
P0123	1:120548005,C>A	missense_SNV	NOTCH2	c.G113T	p.C38F
	4:153247303,T>C	missense_SNV	FBXW7	c.A971G	p.H324R
P0909	4:153244155,TC>T	frameshift_deletion	FBXW7	c.1473delG	p.G491fs
	9:139395150,T>C	missense_SNV	NOTCH1	c.A5788G	p.T1930A

Table S2 Top 20 genes of each CD8$^+$ T cell cluster

CD8$^+$ T cells cluster name	Top 20 genes
0	*CXCR4, SRGN, TNFAIP3, CREM, YPEL5, LMNA, PDE4B, DUSP2, ZFP36, TSC22D3, RGCC, RNF19A, TUBA4A, DDIT4, ANXA1, HERPUD1, CCL4, DCTN6, CRTAM, GPR183*
1	*GPR15, TMIGD2, CD160, ITGA1, FOS, KLRC2, CD69, CAPG, PTGER4, FOSB, KLRC3, NR4A2, HOPX, KLRC1, XCL1, SMIM3, MAP3K8, NFKBIZ, BRE-AS1, XCL2*
2	*S1PR1, LINC00861, RIPOR2, TXNIP, RPS14, GIMAP7, PLAC8, SELL, RPL34, FCMR, RPLP2, IL7R, GIMAP1-GIMAP5, GZMK, GIMAP5, KLRG1, RPL36, CCR7, CMC1, S100A4*
3	*FGFBP2, CX3CR1, S1PR5, FCGR3A, NKG7, FGR, PLEK, GZMH, ADGRG1, FCRL6, PRF1, S100A4, ITGB2, PLAC8, PATL2, KLRG1, TTC38, ADRB2, GNLY, C1orf162*
4	*CXCL13, HAVCR2, CCR1, TNFSF4, CXCR6, MIR155HG, PDCD1, CD82, OAS1, CD27, APOBEC3C, CD27-AS1, CD2, APOBEC3G, IFI44, MIR155, IFI6, ACP5, CCL3, GZMB*
5	*PCLAF, ZWINT, CXCL13, HAVCR2, TK1, MIR3917, TYMS, KRT86, RRM2, CD82, GEM, CCL3, NUSAP1, TNFRSF18, STMN1, VCAM1, MCM7, PCNA-AS1, HMGB2, TUBA1B*
6	*HSPA1L, HSPA1A, HSPA1B, DNAJB1, HSP90AA1, HSPA8, DNAJA1, HSP90AB1, EGR1, HSPE1, HSPH1, JUN, HSPD1, NR4A1, BAG3, DNAJB4, ZFAND2A, HSPA6, HSPB1, IFNG*
7	*SNORD27, SNORD28, SNORD26, SNORD29, SNORD25, SNORD30, SNORD22, SNORD31, SNHG1, CXCR4, ZFP36, ZNF331, TSC22D3, LMNA, SNORA44, TUBB4B, SNORA61, TOB1, DCTN6, SNORD99*

Table S3 Top 20 genes of each Treg cell cluster

Treg cells cluster name	Top 20 genes
0	*DNAJB1, DUSP1, JUNB, HSP90AA1, FOS, NR4A2, PPP1R15A, HSPA1A, DNAJA1, PMAIP1, BTG2, CXCR4, EGR1, JUN, HSPE1, TOB1, RGS2, SNORD22, CD69, DNAJB4*
1	*TNFRSF4, TNFRSF18, TNFRSF9, PKM, ZBTB32, CDKN1A, CD83, IL1RN, LDHA, ICOS, SYNGR2, IL2RA, IL1R2, NFKBIA, NINJ1, EBI3, LGALS1, PTP4A3, NFKBID, GNLY*
2	*OAS1, FOXP3, HLA-DQA1, HLA-DRA, SLAMF1, CXCR3, CD74, MX1, PIK3IP1, IL2RB, STAT1, SLA, HLA-DRB6, IDH2, CISH, CXCR6, IFI6, C3AR1, LY6E, RTP4*
3	*AES, PI16, CD52, RIPOR2, LIME1, GIMAP4, TXNIP, LEF1, GIMAP7, TRAF3IP3, LDHB, MAL, BIN2, GSTK1, GIMAP1-GIMAP5, SELL, ITGB1, RNF214, GIMAP5, TRAT1*
4	*GPR25, IL17A, IL17F, GPR34, CTSH, TMIGD2, MIR5690, KLRB1, TMSB4X, LINC00892, CD40LG, JAML, GIMAP5, GZMA, GIMAP1-GIMAP5, CD52, CAPG, ALOX5AP, GZMB, CCL5*

Table S4 Somatic mutations of notch pathway related genes in TCGA cohort

	all	single	Ratio(all/526)
No mutation	368	-	69.9%
Notch1 mutation	22	9	4.2%
Notch2 mutation	25	3	4.8%
Notch3 mutation	32	10	6.1%
Notch4 mutation	24	7	4.6%
Adam10 mutation	17	8	3.2%
Adam17 mutation	13	3	2.5%
γ-secretase mutation	29	9	5.5%
Numb mutation	9	1	1.7%
Fbxw7 mutation	92	61	17.5%
